# Serological Evidence of Henipavirus Exposure in Cattle, Goats and Pigs in Bangladesh

**DOI:** 10.1371/journal.pntd.0003302

**Published:** 2014-11-20

**Authors:** Sukanta Chowdhury, Salah Uddin Khan, Gary Crameri, Jonathan H. Epstein, Christopher C. Broder, Ausraful Islam, Alison J. Peel, Jennifer Barr, Peter Daszak, Lin-Fa Wang, Stephen P. Luby

**Affiliations:** 1 International Centre for Diarrheal Diseases Research, Bangladesh (icddr, b), Dhaka, Bangladesh; 2 CSIRO Australian Animal Health Laboratory (AAHL), Geelong, Victoria, Australia; 3 EcoHealth Alliance, New York, New York, United States of America; 4 Uniformed Services University, Bethesda, Maryland, United States of America; 5 Environmental Futures Research Institute, Griffith University, Nathan, Queensland, Australia; 6 Program in Emerging Infectious Diseases, Duke-NUS Graduate Medical School, Singapore; 7 Stanford University, Stanford, California, United States of America; University of Texas Medical Branch, United States of America

## Abstract

**Background:**

Nipah virus (NiV) is an emerging disease that causes severe encephalitis and respiratory illness in humans. Pigs were identified as an intermediate host for NiV transmission in Malaysia. In Bangladesh, NiV has caused recognized human outbreaks since 2001 and three outbreak investigations identified an epidemiological association between close contact with sick or dead animals and human illness.

**Methodology:**

We examined cattle and goats reared around *Pteropus* bat roosts in human NiV outbreak areas. We also tested pig sera collected under another study focused on Japanese encephalitis.

**Principal Findings:**

We detected antibodies against NiV glycoprotein in 26 (6.5%) cattle, 17 (4.3%) goats and 138 (44.2%) pigs by a Luminex-based multiplexed microsphere assay; however, these antibodies did not neutralize NiV. Cattle and goats with NiVsG antibodies were more likely to have a history of feeding on fruits partially eaten by bats or birds (PR = 3.1, 95% CI 1.6–5.7) and drinking palmyra palm juice (PR = 3.9, 95% CI 1.5–10.2).

**Conclusions:**

This difference in test results may be due to the exposure of animals to one or more novel viruses with antigenic similarity to NiV. Further research may identify a novel organism of public health importance.

## Author Summary

Nipah virus (NiV), is an emerging disease that causes severe encephalitis and respiratory illness in humans. Pigs were identified as an intermediate host for NiV transmission in Malaysia, and in Bangladesh three NiV outbreak investigations since 2001 identified an epidemiological association between close contact with sick or dead animals and human illness. We collected samples from cattle and goats reared around *Pteropus* bat roosts in human NiV outbreak areas in Bangladesh, and tested pig sera collected for a Japanese encephalitis study. We detected antibodies against NiV glycoprotein in 26 (6.5%) cattle, 17 (4.3%) goats and 138 (44.2%) pigs by a Luminex-based multiplexed microsphere assay, but none were virus neutralizing. There may have been exposure of Luminex positive animals to one or more novel viruses with antigenic similarity to NiV. Further research may identify a novel organism of public health importance.

## Introduction

Nipah virus (NiV) is a zoonotic paramyxovirus whose reservoir host is fruit bats of the genus *Pteropus* [1]–[3]. NiV was first recognized in a large outbreak in Malaysia where pigs were an intermediate host for the transmission of NiV infection in humans [4], [5]. Outbreak investigators speculated that pigs were infected with NiV by ingesting partially eaten saliva-contaminated fruit dropped by *Pteropus* bats [6]. Pig farmers were more likely to be infected with NiV suggesting infected pigs transmitted NiV to humans through close contact [7]. Between 2001 and 2013 NiV has caused 227 recognized human infections in Bangladesh with a case fatality of over 75% [8]–[15]. Although there is no serological or microbiological confirmation of NiV infection in domestic animals in Bangladesh, three outbreak investigations have identified suggestive associations between domestic animals and human infection. In the 2001 outbreak in Meherpur, Bangladesh, human Nipah cases were 7.9 times more likely than controls to have contact with a sick cow (odds ratio[OR] 7.9, 95% confidence interval [CI] 2.2–27.7) [8]. In a 2004 outbreak, a NiV-infected child had a close contact history with two sick goats and in a 2003 human Nipah outbreak at Naogaon, Bangladesh, cases were more likely than controls to have had contact with a nomadic pig herd (OR 6.1, 95% CI 1.3–27.8) [16], [17]. Bats frequently visited date palm trees and licked shaved surfaces of the trees to drink sap at night [18]. Date palm sap spoiled by bat feces is occasionally fed to cattle in Bangladesh [19]. Domestic animal infection with NiV may represent an immediate risk to human infection as well as a risk for further evolution of the virus for adaptation to mammals other than bats. We conducted a cross-sectional study to look for evidence of NiV antibodies in domestic livestock, including cattle, goats and pigs, and to identify exposures associated with NiV antibodies.

## Materials and Methods

### Ethical statement

Field staff obtained written consent from the animal owners for data and sample collection. icddr, b's Research Review Committee, Ethical Review Committee and Animal Experimentation Ethics Committee reviewed and approved the study protocols. The protocol numbers are PR-10015 for the henipavirus study and 2008–063 for the Japanese encephalitis study.

### Study site

For assessing NiV exposure in cattle and goats, we selected Faridpur, Rajbari, Meherpur, Tangail and Naogaon districts as study sites because they had previous human NiV outbreaks. We identified the nearest *Pteropus* bat roost from the human index case's household for each of the five sites. We enrolled cattle and goats living within a 1000 meter radius of the fruit bat roost in each site. If an insufficient number of cattle and goats were identified, we extended this area up to 5000 meters in increments of 1000 meters. We enrolled the pig samples from a population based survey done in pigs in 3 adjacent Northwestern districts (Naogaon, Rajshahi and Nawabganj) of Bangladesh during May-September 2009 as part of a separate study on Japanese encephalitis [20]. Those three districts were chosen for pig sampling because of higher number of Japanese encephalitis cases reported from these areas [21].

### Animal enrollment

For cattle and goat enrollment, we generated random latitude/longitude coordinates within a 1000 meter radius of each of the five selected *Pteropus* bat roosts using global positioning system (GPS) coordinates. From each GPS location, we identified the nearest household. For selecting subsequent households, we chose the nearest front door of every second household. We enrolled a maximum of three animals, either cattle or goats or both, that were either healthy or sick from each household. We selected animals aged >2 months or when they were weaned from the dam's milk and could feed on grass or other foods in the environment that may be contaminated with henipaviruses. For pig specimens the study team conducted a census of the pig population at Naogaon, Rajshahi and Nawabganj districts relying on the pig raisers' social network [22]. The primary objective of the pig sampling was a separate study exploring prevalence of infection with Japanese encephalitis virus, and as a result the field team did not collect the same information on fruit bat exposure as was collected for cattle and goats. Field workers visited the areas to collect data on demographics and management of pigs and sampled 312 pigs. The study team selected pigs over 6 months of age for sample collection because of their exposure to Japanese encephalitis virus for longer period.

### Data collection

Field staff interviewed animal owners to collect information on their animal characteristics, management, ecological and environmental data using a structured questionnaire. The management data included rearing systems and feeding practices. We categorized feeding practices for cattle and goats as intensive (animals are kept in pens and supplied feed entirely from outside), semi-intensive (sometimes grazing and sometimes supplied feed in pens) and extensive (only grazing without supplementation). For pigs, field staff collected rearing system data on two categories including backyard (pigs were allowed to graze in the nearby pasture) and nomadic (pigs were allowed to move from one area to another for scavenging feed).

### Sample collection and laboratory testing

We collected five to eight ml of blood for preparing serum from each selected cattle, goat or pig using aseptic sterile equipment. All animal sera were tested at the Australian Animal Health Laboratory (AAHL) using a Luminex-based multiplexed microsphere assay that specifically detects antibodies to the soluble attachment glycoproteins (sG) of henipaviruses (NiV and Hendra virus (HeV)) [23]. Beads coated with either NiVsG or HeV sG were mixed with sera at a dilution of 1∶100. Biotinylated Protein A/G and Streptavidin-PE were then used to detect bound antibody. Beads were interrogated by lasers in a BioRad BioPlex machine and the results recorded as the Median Fluorescent Intensity (MFI) of 100 beads. Bayesian mixture models were used to characterize the bimodal distribution of microsphere assay outputs to classify individuals as seropositive or seronegative, following methods described in Peel et al. [24]. In contrast to Peel et al (2013), where similar results were obtained whether mixture models were fitted to data from different age groups within the one species simultaneously or independently, for the data from different species described here, optimal fitting was observed when each species was fitted independently. Conservative species-specific cutoffs were determined so that individuals with MFI values above this cutoff were >99% likely to be seropositive (MFI  = 300 for cattle and goats and MFI  = 650 for pigs). Full details of the method, assumptions and results are provided in the Supporting Information to this manuscript. Cattle, goat and pig sera showing higher MFI values were further analyzed by western blot (WB), enzyme-linked immunosorbent assay (ELISA) and serum neutralization test (SNT). The WB test was used to detect non-neutralizing antibodies against recombinant N protein of henipaviruses [25]. A subset of NiVsG positive sera were also tested against Cedar virus (CedV) sG in the Luminex assay. Laboratory personnel at the Viral Special Pathogens Branch, Centers for Disease Control and Prevention tested all NiVsG positive sera, along with a randomly selected a subset of negative sera using their in-house enzyme-linked immunosorbent assay (ELISA). Gamma-irradiated lysates from NiV-infected and mock-infected Vero E6 cells were used as antigens and Protein A/G used for detection of bound antibodies [26]. SNT was performed at AAHL under biosafety level (BSL) 4 conditions. Briefly, sera diluted 1∶10 was mixed with 200 TCID_50_ NiV in 96-well tissue culture plates, incubated for 30 minutes at 37°C and 100 ul containing 2×10^4^ vero cells in suspension added. The cells were incubated for 3 days and then observed for viral CPE.

### Statistical analysis

We calculated the prevalence of antibodies separately for cattle, goats and pigs by dividing Luminex-positive animals by the total number of animals of that species tested. We calculated the prevalence ratio (PR) to identify the association between Luminex results and exposure variables by bivariate analysis. Before examining the independence of multiple explanatory variables, we framed a causal diagram to identify causal associations between variables of interest and to identify confounders as described [27], [28]. Exposure variables having a prevalence ratio >1 in bivariate analysis and selected variables from the causal diagram were entered to construct the final model of multivariate logistic regression analysis. We adjusted all confidence limits for geographical clustering in both bivariate and multivariate logistic regression model to minimize clustering effect during animal enrollment. Based on geographical position of enrolled households, district wise cluster was formed with unique code. Confounding variables were also entered in the multivariate logistic regression model for adjustment during analysis. All statistical analysis was done by using STATA 10.0.

## Results

### Demographic characteristics

We enrolled 400 cattle, 400 goats and 312 pigs between May 2009 and January 2011. Among all enrolled cattle and goats, 798 (99%) were reared in backyard farms, 587 (73%) cattle and goats were fed using semi-intensive practices, 150 (19%) were fed using intensive practices, and 63 (8%) were fed using extensive practices. The median age of sampled cattle was 33 months; 67% were female and 46% were a local breed. The mean age of sampled goats was 21 months; 69% were female and 94% were Black Bengal breed. The study team identified 5,450 households rearing a total of 11,364 pigs throughout Rajshahi (34%), Nawabgonj (13%) and Naogaon (53%) districts. More than 60% (n = 6,963) of pigs were over 12 months of age and half of the total pig population were female. Of the 312 sampled pigs, 49% were female and all were a local breed. The mean age of sampled pigs was 23 months (range 5–60).

### Serological analysis

**Figure 1:**
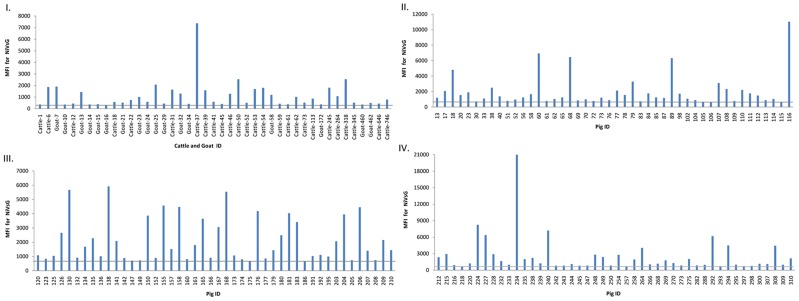
Detection of NiVsG antibodies in Luminex based multiplexed microsphere assay. The median fluorescent intensities (MFI) for each microsphere population are shown in graphs. MFI for antibody positive cattle and goat shown in graph I. MFI for antibody positive pig sera is shown in graph II, III and IV. The gray bar represents the detection cut-off of 300 MFI for cattle and goat sera and 650 MFI for pig sera.

**Figure 2:**
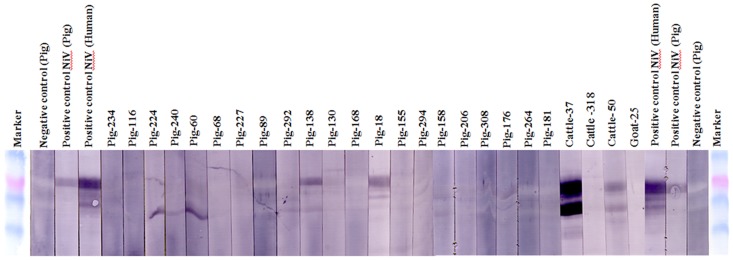
Western blot analysis against NiV N (nucleocapsid) protein of cattle, goat and pig sera showing higher MFI. The marker is BenchMark Pre-stained Protein Ladder (Invitrogen); the positive sera (NiV virus neutralization test positive pig and human field sera); the negative control sera (NiV virus neutralization test negative pig).

Of the tested animals, 26 cattle (6.5%, 95% CI 4.3–9.4), 17 goats (4.3%, 95% CI 2.5–6.7) and138 pigs (44.2%, 95% CI38.6–49.9) had antibodies against NiV soluble attachment glycoproteins (NiVsG) in the Luminex assay (Table 1). The NiVsG positive sera had a range of MFI values between 306 and 20,975 (Figure 1). A total of 39 NiVsG positive sera (9cattle, 2 goats and 28 pigs) showing the highest MFI in Luminex assay were further tested by serum neutralization test against NiV. No neutralizing antibodies were detected. We also tested NiVsG positive sera from 3 cattle, 1 goat and 21 pig sera that reacted most strongly in Luminex assay by western blot. Antibodies against NiV N protein were detected in two cattle sera, one with an MFI value of 7365 and one with an MFI of 2537 and two pig sera (Figure 2). NiVsG positive sera along with 140 NiVsG negative sera (9 cattle, 13 goats and 118 pigs) were tested for NiV antibodies using CDC's in-house ELISA. All specimens were negative for NiV antibodies by ELISA. A total of 25 NiVsG positive sera were tested for CedV antibodies in the Luminex assay. None showed significant binding for CedV.

**Table 1:** NiVsG seropositive animals as detected by Luminex assay.

Species	Number of animals tested	Number NiVsG positive (%)
Cattle	400	26 (6.5)
Goat	400	17 (4.3)
Pig	312	138 (44.2)

**Figure 3:**
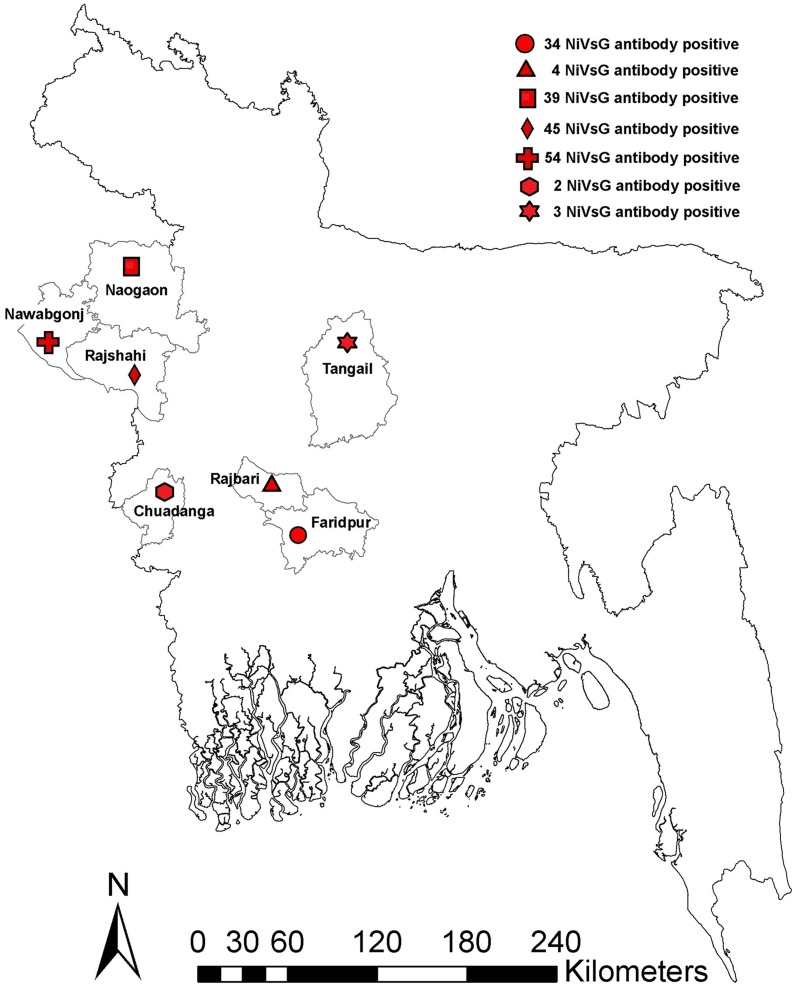
Distribution of NiVsG antibody positive animals in seven districts of Bangladesh.

We identified NiV Luminex antibody positive animals from all study sites (Table 2 and Figure 3). The majority of NiV antibody positive cattle (92%) and goats (94%) were female (Table 3). During sample collection, 99% of animals were observed to be apparently healthy and all antibody positive animals had no apparent clinical signs of illness.

**Table 2:** NiVsG seropositive animals by district in Bangladesh.

Area (District)	Number of animals tested			Number NiVsG positive (%)
	Cattle	Goat	Pig	
Faridpur	80	80	0	34 (21.3)
Rajbari	80	80	0	4 (2.5)
Tangail	80	80	0	3 (1.9)
Chuadanga	80	80	0	2 (1.3)
Naogaon	80	80	109	39 (14.5)
Rajshahi	0	0	100	45 (45)
Nawabganj	0	0	103	54 (52.4)

**Table 3:** Demographic characteristics of NiVsG seropositive and seronegative cattle, goats and pigs in Bangladesh.

**Cattle**	**NiVsG positives (N = 26)**	**NIVsG negatives (N = 374)**
Mean age in month (SD)	32.3 (20)	32.8 (32)
**Sex, no. (%)**		
Female	24 (92)	242 (65)
Male	2 (8)	132 (35)
**Breed, no. (%)**		
Local (indigenous)	19 (73)	166 (44)
Crossbred	7 (27)	208 (56)
**Goat**	**NiVsG positives (N = 17)**	**NiVsG negatives (N = 383)**
Mean age in month (SD)	27.2 (12.8)	21 (18.2)
**Sex, no. (%)**		
Female	16 (94)	260 (68)
Male	1 (6)	123 (32)
**Breed, no. (%)**		
Black Bengal	16 (94)	359 (94)
Jamunapari	1 (6)	18 (5)
Crossbred	0	6 (1)
**Pig**	**NiVsG positives (N = 138)**	**NiVsG negatives (N = 174)**
Mean age in month (SD)	22.3 (10.8)	23.3 (10.4)
**Sex, no. (%)**		
Female	68 (49)	85 (49)
Male	70 (51)	89 (51)
**Breed, no. (%)**		
Native	138 (100)	174 (100)

### Animal management practices and environmental exposures

In bivariate analyses, cattle and goats with NiVsG antibody levels above the chosen cutoffs were more likely to have a history of being fed partially bat and/or bird eaten-fruits (PR = 3.9, 95% CI 2–7.2, p<0.001), drinking raw juice prepared from bat and/or bird-eaten Asian Palmyra palm fruits (*Borassus flabellifer*) (PR = 9.5, 95% CI 5.2–17.4, p<0.001), grazing in areas exposed to roaming pig herds (PR = 1.7, 95% CI 0.6–4.3, p = 0.3), and living in fruit orchard areas (PR = 1.7, 95% CI 0.8–3.8, p = 0.2) (Table 4). However, in multivariate analysis the two exposures that were independently associated were having a history of feeding on fruits partially eaten by bats or birds (PR = 3.1, 95% CI 1.6–5.7, p = 0.001) and drinking of raw palmyra palm juice (PR = 3.9, 95% CI 1.5–10.2, p = 0.004) (Table 5). Out of 800 cattle and goats, 2% (n = 16) of animals were fed juice prepared from partially bats and/or birds-eaten Asian Palmyra palm fruit by their owners. There was no significant difference in pig NiVsG seroprevalence between backyard and nomadic rearing systems (20% in backyard vs. 15% in nomadic herds, p = 0.4).

**Table 4:** Bivariate analysis of feeding practices and environmental exposures associated with NiV serology results in cattle and goats.

Variables	NiVsG positives (N = 43)	NIVsG negatives (N = 757)	PR* (95% CI)	*P*
**Feeding exposures, no. (%)**				
Feeding of partially bat and/or bird eaten fruits	25 (58)	187 (25)	3.9 (2–7.2)	<0.001
Drinking of raw palmyra palm juice	7 (16)	9 (1)	9.5 (5.2–17.4)	<0.001
**Feeding system, no (%)**				
Intensive	0	63 (8)		
Semi-intensive and extensive	43 (100)	694 (92)	undefined	<0.001
**Environmental exposures**				
Roaming pig herds within one km radius area from animal household in last one year, no. (%)	39 (91)	645 (85)	1.7 (0.6–4.3)	0.3
Fruit orchards within one km radius area from animal household, no. (%)	20 (47)	250 (33)	1.7 (0.8–3.8)	0.2
Mean distance in meters between animal household and bat roost (SD)	381 (256.2)	475.1 (296.2)	-	-

*prevalence ratio.

**Table 5:** Multivariate logistic regression analysis of feeding practices and environmental exposures associated with NiV serology results in cattle and goats.

Variables	Adjusted PR*	95% CI	*P*
Feeding of partially bat and/or bird eaten fruits	3.1	1.6–5.7	0.001
Drinking of raw palmyra palm juice	3.9	1.5–10.2	0.004
Fruit orchards within one km radius area from animal household	1.6	0.8–3.3	0.2
Roaming pig herds within one km radius area from animal household in last one year	1.9	0.6–6.4	0.29

*prevalence ratio adjusted for outbreak districts, bat roost distance, feeding systems and rearing system.

## Discussion

This study identified antibodies against NiVsG in 26 cattle, 17 goats and 138 pigs; however these antibodies did not neutralize NiV, and did not react against NiV antigens in an ELISA, though 2 cattle and 2 pig sera reacted with NiV N protein by WB. Animals that were fed fruit that had been partially eaten by bats or birds were >3 times more likely to have antibodies against NiVsG compared with animals not fed partially eaten fruit.

The serological response in these domestic animals suggests they were likely infected with a henipavirus. The positive test results on two different diagnostic platforms targeting two different NiV proteins (sG and N), but negative SNT results and the association with bat bitten fruit suggests that the animals were likely infected with a non-Nipah henipavirus. Cedar virus (CedV) is the only non-Nipah non-Hendra henipavirus to have been isolated and fully described [29], yet there is evidence of considerable diversity of henipaviruses. Samples from 6 bat species in 5 different African countries identified RNA sequence of paramyxovirus L gene suggestive of 19 novel non-Nipah non-Hendra henipaviruses [30]. Three additional novel henipaviruses have been identified by sequencing nucleic acid of the paramyxovirus large gene from *Pteropus giganteus*, the putative bat reservoir of NiV in Bangladesh [31].The virus (or viruses) detected here appear to be more closely related to NiV than HeV, as measured by cross-reactive antibodies specific for NiVsG.

Phylogenetic analysis of NiV isolates from Malaysia and Bangladesh suggest that strains of NiV transmitted from bats to humans were genetically diverse, however all isolated viruses from animals and humans in these two countries show full cross-neutralizing antibodies [32]–[34]. While studies on African bats have showed antigen-antibody reactions to henipaviruses in the Luminex assay, and cross-neutralization of HeV and NiV in serum neutralization tests [35], [36], studies in Vietnam on bats and in Ghana on pigs showed similar types of antigen-antibody reactions of henipaviruses in the Luminex assay without cross neutralization, similar to what we identified in domestic animals in Bangladesh [37], [38]. Cedar virus, detected in Australian fruit bats, is also not cross-neutralizing with HeV or NiV and has limited cross-reactivity in the Luminex sG binding assays [29]. Finally, in India some individual *Pteropus* bats have shown antibodies that cross-neutralized Nipah and Hendra virus [39]. Taken together these observations suggests that there is a spectrum of henipavirus strains circulating, with differing levels of antibody cross-reactivity. Challenges associated with assessing serological responses to an uncharacterized virus were mitigated here by using a Bayesian mixture model approach, which enables the assay output to be assessed in its own right, without the need to compare it to an alternative assay [24]. These analyses strongly supported cutoffs of MFI  = 300 for cattle and goats and MFI  = 650 for pigs as being very conservative (individuals >99% likely to be seropositive) (Details in the supporting material). Fruit bats can contaminate fruits, grasses or other plants with henipaviruses through their excretions and secretions. Epidemiological findings from multiple HeV outbreaks in Australia suggested that the horse index cases were likely to have been exposed via feeding in paddocks containing fruit trees frequented by fruit bats and thereby contaminated with HeV [40]. In our study, animal owners reared animals mainly in the backyard (≈99%) and 73% of these animals were fed with a semi-intensive feeding system. Pteropid bats visit fruit trees as part of their nightly foraging activities, and sometimes drop partially eaten fruits to the ground [41], [42]. Nipah virus RNA has been detected from urine and throat swab samples collected from *P. giganteus* in Bangladesh [31] from fruit partially eaten by *P. hypomelanus* and *P. vampyrus* in Malaysia [41]. As the domestic animals in this study were scavenging for a portion of their daily feeding time, they could have been exposed to dropped fruits or an environment contaminated with bat excreta, which might increase the risk of henipa-like virus transmission from bats to these animals.

In this study, animal owners reported that sometimes they offered dropped fruits as foods to their animals. A few animal owners also reported that they prepared fresh juice from intact Asian Palmyra palm fruit for themselves and they used Palmyra palm fruits partially eaten by bats and/or birds for their animals. The association between exposure to bat-contaminated feeding exposure and presence of antibodies detected by Luminex assay against NiVsG proteins in livestock animals suggests that *P. giganteus* bats, the reservoir species of NiV, or a related frugivorous bat species such as *Cynopterus sphinx* or *Rousettus leischenaulti* – both common in Bangladesh and observed to have similar foraging patterns with *P. giganteus* [43], could be the source of infection that resulted in the generation of these antibodies.

Henipaviruses can infect a wide variety of animal species including humans [4], [44]–[48]. This is consistent with the ability of the virus to infect a wide range of mammals by exploiting the very well conserved ephrin B2 and ephrin B3 receptor [49]–[51]. In Malaysia, antibodies against NiV were detected in goats, dogs, cats and horses during a human Nipah outbreak that suggests a wide range of animal species were exposed and infected with NiV [44]–[46]. Pigs were identified as the most frequently infected domestic animal hosts and they transmitted infection from bats to humans as an intermediate host [4], [5]. In this study, our data also suggest pigs were more likely to be exposed to henipaviruses than cattle and goats. The high rate of seropositivity in pigs could be due to the frequent exposure and/or their high susceptibility to henipavirus infection. Alternatively, this may represent a henipavirus that has adapted to and developed a reservoir in swine. Swine in Malaysia and in Ghana have evidence of susceptibility to henipavirus infection [5], [38]. We don't know whether other henipaviruses are infecting human populations, but further investigation in bats, domestic animals and people may further clarify henipavirus ecology in Bangladesh and globally.

This serological study of healthy animals provides little insight on the clinical consequences of these infections. All antibody positive animals were apparently healthy during sample collection, but they may have had signs of disease earlier. Moreover, animals with severe illness may have died before sampling. Further studies in sick animals would be necessary to evaluate the association of these non-Nipah henipaviruses with illness.

Laboratory findings suggest cattle, goats and pigs were exposed to a novel virus or viruses with antigenic similarity to NiV. The association of antibody positive findings by Luminex assay in cattle and goats with exposures to potentially bat-contaminated foods suggests that the source of this virus is likely frugivorous bats. Further research should be undertaken to characterize the range of henipaviruses spilling over from bats to domestic animals because of their potential animal health and human health importance.

## Supporting Information

Supporting Information S1 Analysis of NiV serological data from domestic species in Bangladesh using Bayesian mixture models.
(PDF)

Supporting Information S2 Questionnaire for serological evidence of henipavirus exposure in cattle and goats.
(PDF)

Supporting Information S3 Datasheet 1.
(XLS)

Supporting Information S4 Datasheet 2.
(XLS)
