# Supplementary material for: Serological Evidence of Henipavirus Exposure in Cattle, Goats and Pigs in Bangladesh
Source: PLoS Negl Trop Dis. 2014 Nov 20;8(11):e3302. doi: 10.1371/journal.pntd.0003302 (PMC4238985; doi:10.1371/journal.pntd.0003302)
Supplement: Supporting Information S1 — Analysis of NiV serological data from domestic species in Bangladesh using Bayesian mixture models. (PDF) [file pntd.0003302.s001.pdf]

ANALYSIS OF NIV SEROLOGICAL DATA FROM DOMESTIC SPECIES IN BANGLADESH USING BAYESIAN MIXTURE MODELS

Supporting information for:
"Serological Evidence of Henipavirus Exposure in Cattle, Goats and Pigs in Bangladesh"
Sukanta Chowdhury, Salah Uddin Khan, Gary Crameri, Jonathan H. Epstein, Christopher C. Broder, Ausraful Islam, Alison J. Peel, Jennifer Barr, Peter Daszak, Lin-Fa Wang, and Stephen P. Luby

Methods:
In Peel et al. (2013) a Bayesian mixture model was used to characterize the bimodal distribution of microsphere assay outputs to classify individuals as seropositive or seronegative. The authors assumed that detection of antibodies when they are present is uniform across individuals within a species, resulting in the same underlying 'seropositive' or 'seronegative' distributions across age classes. Differences in the observed distributions among age classes or populations are therefore explained solely by changes in proportions seropositive/seronegative.

To extend this to a multi-species study, comparable assumptions must also be made that the detection of antibodies when they are present and the fluorescence produced by a given amount of antibody are independent of species. Following the methods described in Peel et al. (2013), analyses were performed on non-pooled cattle, goat and pig data (Table S1), enabling this assumption to be tested and optimal cutoff values for each species to be determined. In addition to samples described in the main text, a total of 145 pig, goat and cattle samples collected during multiple NiV outbreak in Bangladesh in 2011, and tested contemporaneously with this study's samples, were also included (Table S1).

Mixture models were firstly fitted to the cattle, goat and pig *ln*(MFI) data simultaneously (assuming they share the same underlying distributions), and secondly to each species separately (allowing the underlying distributions to vary among species).

Table S1: Sample details, including species, study description and whether analyses were performed on individual or pooled samples

Study description	Sample analysis subset	Pigs	Cattle	Goat	Included in mixture model?
JE pig survey	Assayed individually	312			Yes
NiV outbreak 2011	Assayed individually	4	82	59	Yes
Targeted study	Assayed individually		42	25	Yes
	Assayed in pools of four only (172 pools)		335	350	No
	Subset of pooled samples repeated individually (12 pools)		23	25	Yes
	**TOTAL**		**400**	**400**	

Results

Firstly, a mixture model was fitted to the cattle, goat and pig *ln*(MFI) data simultaneously. The model convergence, autocorrelation and the Monte Carlo error for the posterior mean were assessed as per Peel et al (2013), and determined as satisfactory. The posterior mean estimates and 95% credible intervals (CI) for the parameters of the fitted model are shown in Table S2. The fitted distributions (Figure S1) indicated good model fit for cattle data, however the seropositive peak was not well captured in goat data and the overall fit was suboptimal for pig data (Figure S1).

Table S2: Posterior means and 95% credible intervals for means and variances of NiV sG binding assay *ln*(MFI) distributions for seronegative animals (μ_N and σ_N^2 and seropositive animals ($\mu_P$and σ_P^2), as well as for the proportions of seronegative (*p*) and seropositive (1-*p*) animals in each species (and the population as a whole), generated from the fitted mixture model, fitted to all species simultaneously (column 1) or to each species separately (column 2-4).

	**ALL**		**Cattle**		**Goat**		**Pig**	
	**Mean**	**95% C.I.**	**Mean**	**95% C.I.**	**Mean**	**95% C.I.**	**Mean**	**95% C.I.**
μ_N	4.8	(4.7-4.8)	4.75	(4.70-4.80)	4.74	(4.68-4.80)	5.20	(5.07-5.36)
μ_P	6.3	(6.2-6.5)	6.15	(5.79-6.53)	5.68	(5.41-5.99)	6.69	(6.5-6.91)
σ_N^2	0.05	(0.03-0.07)	0.04	(0.03-0.06)	0.03	(0.01-0.05)	0.07	(0.02-0.16)
σ_P^2	1.1	(0.9-1.3)	1.00	(0.63-1.50)	0.59	(0.37-0.86)	1.00	(0.8-1.2)
p_{Cattle}	0.70	(0.60-0.78)	0.67	(0.57-0.76)				
$1 - p_{Cattle}$	0.30	(0.22-0.40)	0.33	(0.24-0.43)				
p_{Goat}	0.64	(0.54-0.75)			0.54	(0.40-0.68)		
$1 - p_{Goat}$	0.36	(0.25-0.46)			0.46	(0.32-0.60)		
p_{Pig}	0.03	(0.00-0.08)					0.21	(0.12-0.33)
$1 - p_{Pig}$	0.97	(0.92-1.00)					0.79	(0.67-0.88)
p	0.32	(0.29-0.36)						
$1 - p$	0.68	(0.64-0.71)						

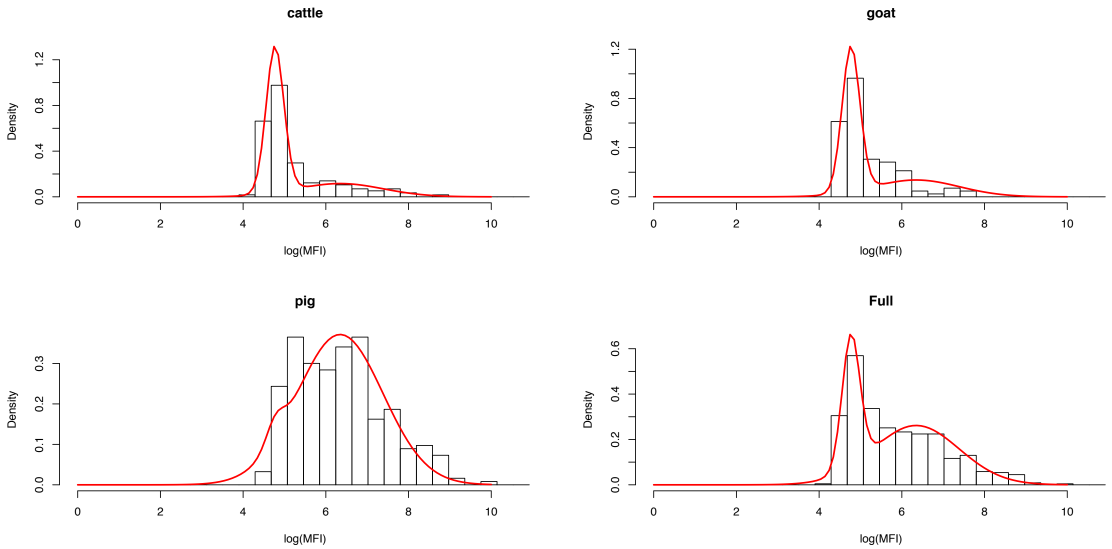

Figure S1: Frequency distribution histograms of NiV sG binding assay *ln*(MFI) values for each species (Cattle: n = 147, Goat: n = 109, Pig: n = 316) and overall. The red lines correspond to the predictive posterior means generated from the fitted mixture model, fitted to all species simultaneously.

The above results were compared to those obtained from a mixture model fitted to each species separately. The number of iterations and burnin were increased from that used in the previous analyses and in Peel et al. (2013) (20,000 and 5,000, respectively) to 100,000 and 50,000, respectively, and resulted in model convergence for all species. The fitted distributions indicated better model fit for each species compared with the previous analysis (Figure S2). Model parameter estimates were similar for cattle and goats (Table S2). Indeed, the mean value for the seronegative distributions were almost identical among analyses (lnMFI= 4.8, 4.75, 4.74 for all data, cattle data and goat data, respectively). Variances were also similar, and the estimated proportions of seronegative and seropositive individuals had closely overlapping credible intervals. In contrast, the mean value for the pig seronegative distribution was shifted to the right (ln(MFI)= 5.2), with a non-overlapping credible interval with cattle and goat estimates, or estimates from all data being fitted simultaneously. Additionally, the estimated proportion seropositive shifted from 97% (CI: 92-100%) to 79% (CI: 67-88%), again, with non-overlapping credible intervals. The mean value for the positive distribution varied across species (*ln*(MFI) = 6.15, 5.68, 6.69 for cattle, goats and pigs, respectively), indicating species differences in the magnitude of antibody responses to henipavirus infection or a insufficiently strong signal in the data.

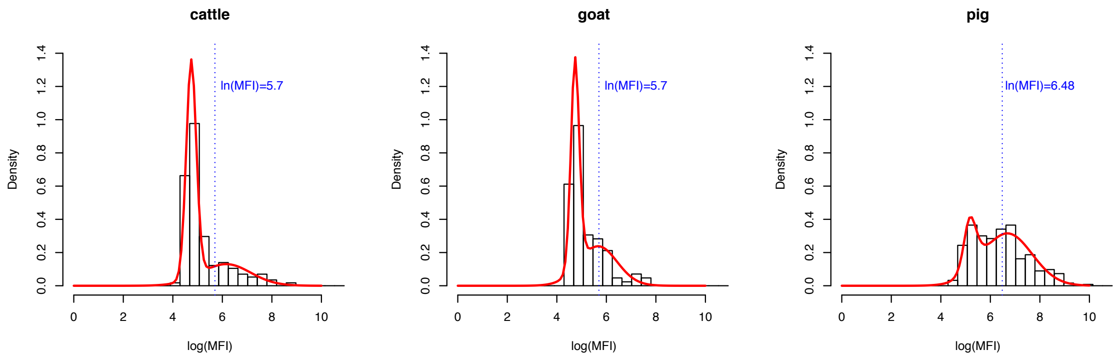

Figure S2: Frequency distribution histograms of NiV sG binding assay *ln*(MFI) values for each species (Cattle: n = 147, Goat: n = 109, Pig: n = 316). The red lines correspond to the predictive posterior means generated from the fitted mixture model, fitted to all species independently. The blue line indicates the location of a cutoff for each species so that individuals with MFI values above this cutoff were >99% likely to be seropositive.

Table S3 provides overall and species-specific estimates for the 'optimal' model cutoff (equally minimizing false negatives and false positives), derived as in Peel et al (2013). The independently fitted models were examined further to assess the probability of belonging to the seropositive or seronegative groups at a range of different cutoffs. The cutoffs illustrated in this analyses were a very conservative cutoff, used in previous analyses (MFI=1500), the 'optimal' cutoff identified for each species (Table S3, MFI = 137, 157, 203), and a cutoff for each species so that individuals with MFI values above this cutoff were >99% likely to be seropositive (Figure S3, Table S4). Based on the mixture model results, individual cattle and goats with an MFI of 300 or above (*ln*(MFI) = 5.7) and individual pigs with an MFI of 650 or above (*ln*(MFI)=6.5) were >99% likely to be seropositive. These cutoffs would produce seroprevalences of 6.5% (CI 4.3 - 9.4%), 4.3% (CI 2.5 - 6.7%) and 44.2% (CI 38.6 - 49.9%) for cattle, goats and pigs, respectively.

Table S3: Posterior means and 95% credible intervals for the optimal cutoff (equally minimizing false negatives and false positives), derived as in Peel et al (2013), for each species (generated from the fitted mixture model, fitted to all species independently).

	**Mean *ln*(MFI)**	**95% C.I.**	**Mean MFI**	**95% C.I.**
Cattle	5.1	(4.9-5.2)	157	(138–186)
Goats	4.9	(4.8–5.1)	137	(121–161)
Pigs	5.3	(5.1–5.6)	203	(161–281)

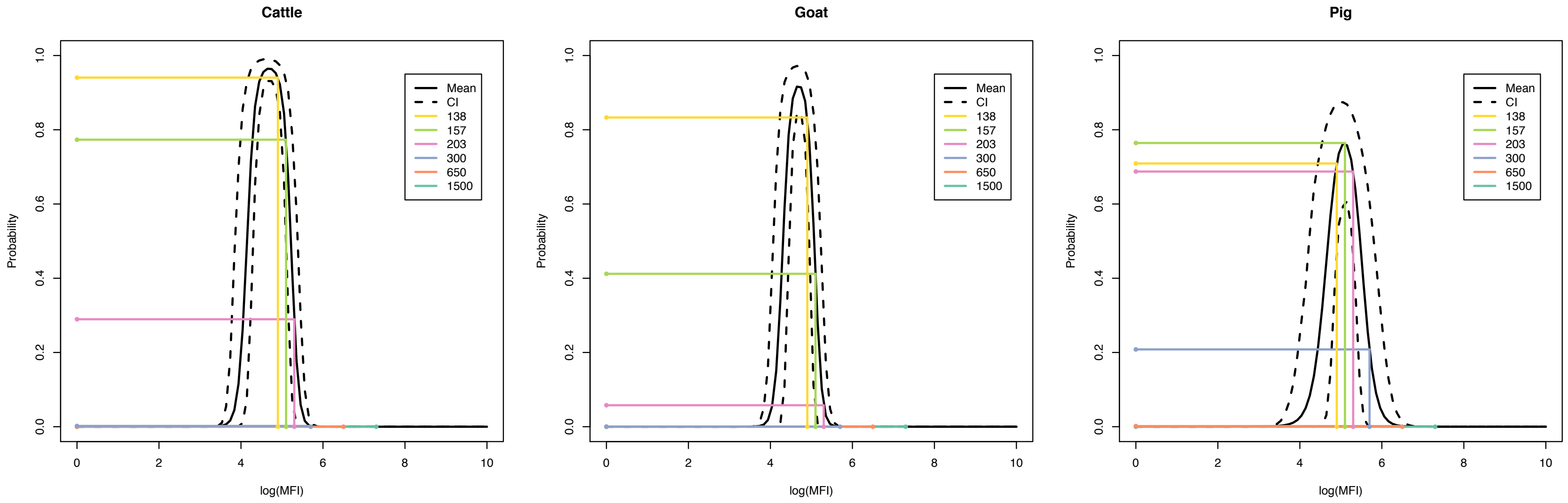

Figure S3: Plots of the posterior mean (and 95% credible intervals) for the relative probability of a given NiV sG binding assay *ln*(MFI) value belonging to the seronegative, rather than seropositive groups for each species (generated from the fitted mixture model, fitted to all species independently). The coloured lines indicate the posterior mean probability of being seronegative (the y-axis intercept) at each of several cutoffs (the x-axis intercept).

Table S4: Posterior means and 95% credible intervals for the probability of belonging to the seronegative group, generated from the fitted mixture model fitted to each species independently and evaluated at different cutoffs.

		Cattle			Goat			Pig		
**MFI**	***ln*(MFI)**	**Mean**	**2.5%**	**97.5%**	**Mean**	**2.5%**	**97.5%**	**Mean**	**2.5%**	**97.5%**
138	4.9	0.94	0.88	0.98	0.83	0.66	0.95	0.71	0.48	0.87
157	5.1	0.77	0.5	0.93	0.41	0.04	0.78	0.76	0.61	0.87
203	5.3	0.29	0.03	0.71	0.06	<0.001	0.29	0.69	0.47	0.83
300	5.7	<0.001	<0.001	0.01	<0.001	<0.001	<0.001	0.21	<0.001	0.61
650	6.5	<0.001	<0.001	<0.001	<0.001	<0.001	<0.001	<0.001	<0.001	0.01
1500	7.3	<0.001	<0.001	<0.001	<0.001	<0.001	<0.001	<0.001	<0.001	<0.001

Discussion

These analyses supported a very conservative cutoff (>99% likely to be seropositive) of MFI = 300 for cattle and goats and MFI= 650 for pigs. The higher cutoff for pigs is a result of a noticeable upward shift in both the pig seronegative and seropositive distributions. Combined with a less clear bimodal shape to the pig data, this finding is supportive of previous reports that henipavirus serological assays on pig samples tend to produce ‘noisy’ results and have generally had to be interpreted with caution (J. Barr, unpublished data, Hayman et al. 2011). Despite this, fitting the data independently to each species allowed appropriate species-specific cutoffs to be determined. As pointed out in Peel et al (2013), “The mixture model approach has the advantage of assessing the microsphere binding assay output in its own right, on a population level, without the need to compare it to an alternative assay with unknown sensitivity and specificity.” In that study, identifiability was improved by combining information from different age classes for model fitting, and did not result in significant differences in parameter estimates to models fitted individually to each age class. The results presented here, i.e. that different results were observed when the model was fitted individually versus to each species separately, imply that this assumption is not valid across species. The fluorescence intensity produced from antibodies when they are present appears to be species-dependent, and further work is required to determine whether this relates to a laboratory artefact, or whether this might reflect inherent differences in immune response to henipavirus infection.

It is worth noting that the range of *ln*(MFI) values reported here is higher than those reported in Peel et al (2013), and represents machine differences. Previous studies have shown a predictable relationship between results from samples tested in duplicate between the two machines (Peel et al, 2013). When a machine-correction factor is applied to previously analyzed data, the mean of the seronegative distribution of cattle and goats is consistent with that identified for African bats.

References

Hayman, D. T. S., Wang, L.-F., Barr, J., Baker, K. S., Suu-Ire, R., Broder, C. C., et al. (2011). Antibodies to Henipavirus or Henipa-Like Viruses in Domestic Pigs in Ghana, West Africa. PLoS ONE, 6(9), e25256. doi:10.1371/journal.pone.0025256

Peel, A. J., McKinley, T. J., Baker, K. S., Barr, J. A., Crameri, G., Hayman, D. T. S., et al. (2013). Use of cross-reactive serological assays for detecting novel pathogens in wildlife: assessing an appropriate cutoff for henipavirus assays in African bats. Journal of Virological Methods, 295–303. doi:10.1016/j.jviromet.2013.06.030
