# Supplementary material for: Serological Evidence of Henipavirus Exposure in Cattle, Goats and Pigs in Bangladesh
Source: PLoS Negl Trop Dis. 2014 Nov 20;8(11):e3302. doi: 10.1371/journal.pntd.0003302 (PMC4238985; doi:10.1371/journal.pntd.0003302)
Supplement: Supporting Information S2 — Questionnaire for serological evidence of henipavirus exposure in cattle and goats. (PDF) [file pntd.0003302.s002.pdf]

Questionnaire for Serological Evidence of Henipavirus Exposure in cattle and goats
রক্তে এন্টিবডি পরীক্ষার মাধ্যমে গরু এবং ছাগলে নিপা ভাইরাসের সংক্রমণ অনুসন্ধানের জন্য প্রশ্ন

Supporting information for:

"Serological Evidence of Henipavirus Exposure in Cattle, Goats and Pigs in Bangladesh"
Sukanta Chowdhury, Salah Uddin Khan, Gary Crameri, Jonathan H. Epstein, Christopher C. Broder, Ausraful Islam, Alison J. Peel, Jennifer Barr, Peter Daszak, Lin-Fa Wang, and Stephen P. Luby

Study ID:<br>সনাক্তকারী নং						Date of interview (dd/mm/yyyy):<br>সাক্ষাতের তারিখ			

Interviewer name:
সাক্ষাত গ্রহণকারীর নাম

Section A: Animal owner's information (সেকশন এ: পশুর মালিক সম্পর্কিত তথ্য)

1. Name of the animal owners (পশুর মালিকের নাম) :
2. Place of Interview (সাক্ষাতের স্থান):
   i. Village (গ্রাম):
   ii. Union (ইউনিয়ন):
   iii. Upazilla (উপজেলা):
   iv. District (জেলা):
3. Phone number (ফোন নং):

**GIS record:**<br>**(জি আই এস তথ্য)**	North<br>উত্তর	East<br>পূর্ব

Section B: Information on domestic animals (সেকশন বি: গৃহপালিত পশু সম্পর্কিত তথ্য)

1. Total animals<br>মোট পশু	Species<br>প্রজাতি		Number<br>সংখ্যা
	a) Cattle<br>গরু	☐	
	b) Goat<br>ছাগল	☐	
	c) Others<br>অন্যন্যা	☐	

2. Number of selected animal species for sample and data collection:<br>রক্ত ও নমুনা সংগ্রহের জন্য বাছাইকৃত পশুর সংখ্যা	a) Cattle<br>গরু	☐	
	b) Goat<br>ছাগল	☐	

3. Breed of selected animal species:<br>বাছাইকৃত পশুর জাত	a) Cattle<br>গরু	Local ☐ দেশী cross ☐ সংকর others ☐ অন্যন্যা

	b) Goat<br>ছাগল	Black Bengal ☐<br>ব্ল্যাক বেংগল	Jamunapari ☐<br>যমুনাপারি	others ☐<br>অন্যন্যা

4. Age of selected animal species:<br>বাছাইকৃত পশুর বয়স	a) 2 months - 12 months<br>২ মাস - ১২ মাস	☐
	b) 13 months - 24 months<br>১৩ মাস - ২৪ মাস	☐
	c) 25 months – 36 months<br>২৫ মাস - ৩৬ মাস	☐
	d) 37 months – 48 months<br>৩৭ মাস - ৪৮ মাস	☐
	e) 49 months – 60 months<br>৪৯ মাস - ৬০ মাস	☐
	f) > 60 months<br>৬০ মাসের বেশী	☐
5. Sex of selected animal species:<br>বাছাইকৃত পশুর লিঙ্গ	a) Male<br>পুরুষ	☐
	b) Female<br>স্ত্রী	☐
6. Health status:<br>স্বাস্থ্যের ধরন	a) Apparently healthy<br>আপাতদৃষ্টিতে সুস্থ	☐
	b) Sick<br>অসুস্থ	☐
7. BCS (Body Condition Score):<br>শারীরিক অবস্থা	a) 1 = emaciated<br>১ = হাড্ডিসার	☐
	b) 2 = thin<br>২ = পাতলা	☐
	c) 3 = average body condition<br>৩ = মোটামুটি ভাল	☐
	d) 4 = heavy condition<br>৪ = মোটা	☐
	e) 5 = fatty<br>৫ = বেশী মোটা	☐

Pleases fill up followings (8) if cattle or goats become sick
যদি গরু/ছাগল অসুস্থ থাকে তবে নীচের (৮) তথ্যগুলো পুরন করুন

8. Clinical findings:<br>প্রাপ্ত লক্ষণ সমূহ	a) Temperature<br>তাপমাত্রা		
	b) Pulse/minute<br>ধমনী স্পন্দন/মিঃ		
	c) Respiration/minute<br>শ্বাস-প্রশ্বাস/মিঃ		
	d) Posture:<br>দাড়ানো অবস্থান	Normal<br>স্বাভাবিক	☐
		Recumbent<br>মাটিতে শায়িত	☐
	e) Respiratory sign:<br>শ্বসনতন্ত্রীয় লক্ষণ	Normal<br>স্বাভাবিক	☐
		Coughing<br>কাশি	☐

		Sneezing<br>সর্দি	☐
		Rales<br>নাক ডাকা	☐
		Others<br>অন্যন্যা	()
	f) Neurological sign:<br>স্নায়ুতন্ত্রীয় লক্ষণ	Normal<br>স্বাভাবিক	☐
		Inco-ordination<br>ভারসাম্যহীনতা	☐
		Circling<br>বৃত্তাকারে ঘুরা	☐
		Convulsion<br>খিচুনি	☐
		Stiffness<br>শক্তহয়ে থাকা	☐
		Paresis<br>অবশ হয়ে যাওয়া	☐
		Others<br>অন্যন্যা	
	g) Others sign:<br>অন্যন্যা লক্ষণ	Diarrheal<br>পাতলা পায়খানা	☐
		Salivation<br>মুখ দিয়ে লালা পড়া	☐
		Other<br>অন্যন্যা	

Section C: Last one year disease history (সেকশন সি: গত এক বছরে রোগের ইতিহাস)

1. Among your cattle and goats, how many deaths occurred in the last year?<br>গত বছরে আপনার পালিত গরু এবং ছাগলের মধ্যে কতগুলো মারা গেছে?	Cattle<br>গরু		
	Goats<br>ছাগল		
2. The last time when your cattle or goats were sick, what signs did they have?<br>শেষবার যখন আপনার গরু এবং ছাগল অসুস্থ হয়েছিল, কি ধরনের লক্ষণ দেখা গিয়েছিল?			
3. Did you seek any advice for treating the animal (s)?<br>পশুগুলোর চিকিৎসার জন্য কোনো ব্যবস্থা নিয়েছিলেন কি?		a) Yes<br>হ্যাঁ	☐
		b) No<br>না	☐
4. How did you treat the animal?<br>পশুগুলোর চিকিৎসা কিভাবে করেছিলেন?		a) By Registered Veterinarian<br>নিবন্ধনকৃত পশু চিকিৎসক দ্বারা	☐
		b) By local village doctor<br>স্থানীয় গ্রাম্য চিকিৎসক দ্বারা	☐
		a) Others<br>অন্যন্যা	☐

Section D: Animal Management history (সেকশন ডি: পশুর ব্যবস্থাপনার ইতিহাস)

1. How do you rear your animals?<br>পশুগুলো কিভাবে পালন করেন?	a) Household<br>পারিবারিকভাবে	☐
	b) Herd/Commercial farming<br>পাল/বানিজ্যিক খামারে	☐
2. What type of animal's feeding methods do you manage commonly? **<br>পশুগুলোকে সাধারনত কোন পদ্ধতিতে খাওয়ানো হয়?	a) Extensive<br>উন্মুক্ত পদ্ধতিতে	☐
	b) Semi-intensive<br>আধা-আবদ্ধ পদ্ধতিতে	☐
	c) Intensive<br>আবদ্ধ পদ্ধতিতে	☐
3. How far distance grazing area from the bat roost?<br>পশুর চারনভুমি বাদুরের আবাসস্থল থেকে কত দুরে?	a) 0-1000 meters / 0-1094 yards<br>০-১০০০ মিটার / ০-১০৯৪ গজ	☐
	b) 1001-2000 meters / 1095-2187 yards<br>১০০১-২০০০ মিটার / ১০৯৫-২১৮৭ গজ	☐
	c) 2001-3000 meters / 2188-3281 yards<br>২০০১-৩০০০ মিটার / ২১৮৮-৩২৮১ গজ	☐
	d) 3001-4000 meters / 3282-4375 yards<br>৩০০১-৪০০০ মিটার / ৩২৮২-৪৩৭৫ গজ	☐
	e) 4001-5000 meters / 4376-5468 yards<br>৪০০১-৫০০০ মিটার / ৪৩৭৬-৫৪৬৮ গজ	☐

** Extensive= free range grazing, Intensive= stall feeding and semi-intensive= mixed type (partial extensive and partial intensive)

<u>Section E: Ecological data (সেকশন ই: পরিবেশ সম্পর্কিত তথ্য)</u>

1. Did you see fruit bats around house hold at night?<br>রাত্রে আপনার ঘরের চারিদিকে বাদুর দেখতে পেয়েছিলেন?	a) Yes<br>হ্যাঁ	☐
	b) No<br>না	☐
2. How many fruit orchards within one kilometre radius area?<br>এক কিলোমিটার ব্যাস এলাকার মধ্যে কতগুলো ফলের বাগান আছে?		
3. Did your animal (s) feed on fruits partially eaten by bats/bird/unknown animals?<br>পশুগুলো বাদুর/পাখি/অজানা পশুর আংশিক খাওয়া কোন ফল খেতে দেখেছিলেন কি?	a) Yes*<br>হ্যাঁ	☐
	b) No<br>না	☐
	c) Don't know<br>জানিনা	☐
*If yes, then how?<br>যদি হ্যাঁ হয়, তবে কিভাবে?	Usual practice ☐<br>সচরাচরভাবে	accidentally ☐<br>আকস্মিক
4. Did your animal (s) drink raw date palm sap?<br>পশুগুলোকে খেজুরের রস খেতে দেখেছিলেন কি?	a) Yes*<br>হ্যাঁ	☐
	b) No<br>না	☐
	c) Don't know<br>জানিনা	☐
*If yes, then how?<br>যদি হ্যাঁ হয়, তবে কিভাবে?	Usual practice ☐<br>সচরাচরভাবে	accidentally ☐<br>আকস্মিক

5. Have any pigs herd or household pigs within one kilometre radius area?<br>এক কিলোমিটার ব্যাস এলাকার মধ্যে পারিবারিকভাবে পালিত শুকর অথবা শুকুরের পাল আছে কিনা?	a) Yes<br>হ্যাঁ	☐
	b) No<br>না	☐

Section F: Sick animal exposure history (সেকশন এফ: অসুস্থ পশুর সংস্পর্শে আসার তথ্য)

1. Have you ever been in contact with sick animals?<br>আপনি কি কখনো অসুস্থ পশুর সংস্পর্শে গিয়েছিলেন?	a) Yes<br>হ্যাঁ	☐
	b) No<br>না	☐
2. Did you use any protective measures when working with sick animals?<br>অসুস্থ পশুর সংস্পর্শে কাজ করার সময় আপনি কোন প্রতিরোধ ব্যবস্থা নিয়েছিলেন?	a) Yes<br>হ্যাঁ	☐
	b) No<br>না	☐
3. What type of protective measures did you take?<br>কি ধরনের প্রতিরোধ ব্যবস্থা নিয়েছিলেন?		

Section G: Public health data (সেকশন জি: মানুষের স্বাস্থ্য বিষয়ক তথ্য)

1. Had any identified human Nipah cases in your family?<br>আপনার পরিবারে কেউ কি নিপা আক্রান্ত ছিল?	a) Yes*<br>হ্যাঁ	☐
	b) No<br>না	☐
*If yes, how many persons?<br>যদি উত্তর হ্যাঁ হয়ে থাকে, তবে কত জন?	a) Deaths<br>মৃত	
	b) Recovered<br>আরোগ্যলাভ	
2. Do any Nipah recovered persons of your family currently have any partial mental and physical disability?<br>আপনার পরিবারে নিপা রোগ হইতে মুক্ত কিন্তু রোগ পরবর্তী আংশিক মানসিক এবং শারীরিক অসুস্থতা আছে এরকম কেউ কি আছে?	a) Yes*<br>হ্যাঁ	☐
	b) No<br>না	☐
*If answer is yes, how many?<br>যদি উত্তর হ্যাঁ হয়ে থাকে, তবে কত জন?		

If any overall comment or information:
যদি অন্য কোন মন্তব্য বা তথ্য থাকে
